# Nano round polycrystalline adsorbent of chicken bones origin for Congo red dye adsorption

**DOI:** 10.1038/s41598-024-57412-4

**Published:** 2024-04-02

**Authors:** Edwin Andrew Ofudje, Khairia Mohammed Al-Ahmary, Ibtehaj F. Alshdoukhi, Mazen Rzeeg Alrahili, Yasar N. Kavil, Saeed Saad Alelyani, Ammar M. Bakheet, Abdullah G. Al-Sehemi

**Affiliations:** 1 Department of Chemical Sciences, Mountain Top University, Ibafo, Ogun State Nigeria; 2 Department of Chemistry, College of Science, University of Jeddah, Jeddah, Saudi Arabia; 3 Department of Basic Sciences, College of Science and Health Professions, King Saud Bin Abdulaziz University for Health Science, King Abdullah International Medical Research Center, Riyadh, Saudi Arabia; 4 Physics Department, School of Science, Taibah University, Janadah Bin Umayyah Road, 42353 Medina, Saudi Arabia; 5 Marine Chemistry Department, Faculty of Marine Sciences, King Abdulaziz University, P.O. Box 80207, 21589 Jeddah, Saudi Arabia; 6 Renewable Environment Company for Environmental Consulting (REC), 21589 Jeddah, Saudi Arabia; 7 ChemEconomy, Non Profit Organization for Environment Protection, 46429 Yanbu, Saudi Arabia; 8 Research Center for Advanced Materials Science (RCAMS), King Khalid University, 61413 Abha, Saudi Arabia; 9 Department of Chemistry, College of Science, King Khalid University, 61413 Abha, Saudi Arabia

**Keywords:** Adsorbent, Chicken, Fish bones, Congo red, Nano-crystalline, Environmental sciences, Chemistry, Nanoscience and technology

## Abstract

Nano round polycrystalline adsorbent (NRPA) of chicken bones origin was utilize as effective adsorbent in Congo red dye removal via aqueous media. The NRPA adsorbent was prepared via thermal decomposition and its structure was investigated with the aids of Transmission Electron Microscopy, Fourier Infrared Spectroscopy (FT-IR), Scanning Electron Microscopy, Energy Dispersive X-ray Analysis (EDX), and X-ray Diffractometer (XRD). A monophasic apatite phase was confirmed from XRD investigation, while functional groups analysis showed that NRPA possessed CO_3_^2−^_,_ PO_4_^3−^ and OH^−^ absorption bands. The maximum adsorption capacities derived from Langmuir isotherm is 98.216 mg g^−1^. From the combined values of n from Freundlich and separation factor (R_L_) of Langmuir models, the adsorption of CR by NRPA is favourable. Thermodynamic values of 5.280 kJ mol^−1^ and 16.403 kJ mol^−1^ K^−1^ were found for ΔH° and ΔS° respectively. The entire values of ΔG° which ranges from − 35.248 to − 459.68 kJ mol^−1^ were all negative at different temperatures. Thus, nano polycrystalline adsorbent of chicken bone origin can serve as excellent adsorbent in Congo red dye removal from waste water.

## Introduction

Water and land are vital natural material resources for the sustenance of some basic human, animal and plants needs. However, human encroachment had resulted in the composition of these natural materials being seriously affected and almost rendering them useless. Effluents discharged from industry into the aquatic environment are among the chief causes of the deterioration of water quality^1–4^. Rapid increase in industrialization leading to urbanization had led to the released of huge quantities of wastewater into the environment, causing pollution^5,6^. The textile industry as well as its dye-containing wastewaters is the major sources of organic pollutants. Report has it that up to 25% of textile dyes during dyeing process is lost, while about 2–20% is released into the environmental as aqueous effluents^6,7^. Discharge of effluents containing dyes into water body is detrimental due to their colours which prevent the flow of oxygen directly into water^3–5^.

As at the moment, reactive dyes which are family of azo type are often sort after in some industries such as the printing and dyeing, fabric, electroplating, plastic and pharmaceuticals^8–10^. Direct dyes such as Congo red (CR) are readily soluble in dispersed water and can be ionized into more toxic anions^9^. Although, Congo red is widely used due to its ease of application, greater dyeing performance and low cost, it contains injurious substances which could pollute the environment and cause danger to plants, animals and human health^8–10^. Congo red is really toxic dye which has great resistance towards conventional techniques in the treatment of wastewater^8,9^.

Continuous consumption of water contaminated with CR could damage the body system such as liver, brain, blood system and cause hematopoiesis which could results in serious health challenges such as breathing difficulties, diarrhea, nausea and vomiting^8–10^. Report has it that CR has carcinogenic effect^9^ and as such, there is urgent need for its elimination from wastewater prior to discharge into water ways.

Recently, many techniques such as photo-catalytic degradation, filtration, and adsorption have been deployed in remediating dyes from contaminated sites^7–11^. Among these techniques, adsorption is popularly considered to be the most appropriate method as a result of its high efficacy and efficiency, very easy to operate, eco-friendly and very cheap to practice^6,7^. The commonly used adsorbent in the adsorption process is activated carbon but its application is restricted as a result of its high cost of production and purity particularly to the common man.

To this end, several materials have been reported for Congo red uptake and some of them are FexCo_3_-xO_4_ nanoparticles^6^, soybean curd xerogels^10^, graphene oxide^11^, rice husk^12^, coffee waste^13^, tunics of the corm of the saffron^14^, cadmium(II) metal–organic frameworks^15^, magnesium aluminium layered double hydroxide^16^, guava leaf-based activated carbon^17^, pine bark^18^, and industrial waste^19^ have been developed. Attallah et al.^20^ used metal hydroxide sludge (MHS) obtained from hot dipping galvanizing plant as adsorbent for removal of CR, Maximum uptake capacity of 40 mg g^−1^ was attained at solution pH of 6.0, Kumar et al.^21^ studied the use of starch/AlOOH/FeS_2_ nanocomposite as excellent adsorbent for the removal of CR from an aqueous solution. Adsorption capacity of 333.33 mg g^−1^ was found to be the maximum.

Zehra et al.^22^ utilized peat as an adsorbent for the adsorption of CR and the maximum adsorption capacity was determined to be 10.1 mg g^−1^ at a shaking time of 1.5 h, and solution pH of 6.4. The choice of fabricating NRPA from chicken bones in this research is because of its nontoxic, availability, and because it is not expensive to prepare. With the surge in the demand to produce low-cost and environmentally friendly adsorbents, with special interest in adsorbents with high adsorption affinity towards various pollutants, it thus, become very imperative to search for alternative approaches for the synthesis of high-quality activated carbon. It is on this premises, that this study aimed at investigating chicken bone derived adsorbent for the removal of Congo red, and by so doing, value chain is added to agro-waste.

Chicken bones (CB) are waste material available in numerous quantities in Nigeria and often constitute environmental nuisance. They are often disposed haphazardly which could lead to the breading of microorganisms due to the presence of organic content and subsequently results in health and environmental challenges. Thus, the use of CB as adsorbent for CR removal will serves as a means of decontaminating the environment and adding values to waste and turning them to useful materials. The adsorption potency of nano-round polycrystalline adsorbent in the uptake of CR dye was tested. Factors like contact time, NRPA dose, pH, and CR initial concentration were investigated in a batch process. Mathematical modeling using isotherms, kinetics and thermodynamic models were performed to give better explanations about the mechanism of the adsorption process.

## Materials and methods

### Materials and adsorbent preparation

Required CR concentration used in this work was obtained from Shanghai Macklin Biochemical Co., Ltd (China). All reagents used are of analytical grade and as such, they were utilized without any purification. The bones of chicken were collected from a local food restaurant and thoroughly cleansed using hot water. Thereafter, the bones were crushed and boiled with 0.1 M NaOH for 15 min and raised in distilled water. The pre-treated chicken bones were oven dried and placed in a furnace at 900 °C temperature for calcination. The product formed was grinded into powder after cooling to room temperature. The fabricated product was labeled nano-round polycrystalline adsorbent (NRPA) and further characterization using different techniques.

### Characterizations

TENSOR 27, series FT-IR spectrometer, (Germany) was deployed to examine the various binding groups available on the adsorbent surface using the KBr pellet method. Mixture of KBr powder and NRPA was done in a mortar and pestle using a ratio of 1:99% and thereafter compressed to form a 2 mm pellet. An X-pert PRO, PANalytical, (Netherland) diffractometer was deployed for the phase and purity assessment of the NRPA powder (wavelength = CuKα1). A Carl Zeiss AG (Supra 55VP) scanning electron microscope was deployed for the adsorbent surface morphology analysis, while chemical configuration of NRPA was done using Energy-dispersive X-ray spectroscopy (EDX). Further imaging was achieved with transmission electron microscope (Tecnai 20 G2 FEI, Netherland) after sonicating dispersed particles of the adsorbent in distilled water. Zero point charge (pH_ZPC_) of NRPA was done with a Zetasizer Nano ZS instrument (Malvern, UK). A Quantachrome NOVA 2200C, (USA) analyzer was utilized to measured pore size, pore volume and surface area of NRPA.

### Batch adsorption process

A magnetic orbital shaker was used to conduct the experiments with the content placed inside 100 mL conical flask. The experiment was done in triplicate and an average value was obtained. Efects of CR concentration was done between 25 and 200 mg L^−1^, NRPA dose of 10 to 60 mg, time of 10 to 160 min and solution pH of 2.0 to 7.0. Briefly, 40 mg of the NRPA was added to 25 mL volume of CR solution. The solution was adjusted to desired value by adding either 0.1 M of NaOH or HCl, after which the content was equilibrated on the orbital shaker. At diverse time intervals, certain amount of CR was withdrawn and analyzed with the aids of Ultraviolet–Visible spectrophotometer at 498 nm wavelength. The equilibrium amount of CR adsorbed by the synthesized NRPA was evaluated using the relationship below:

1 $$ Q_{e} = \frac{{C_{o} - C_{e} }}{m} \times V $$

Given that C_o_ and C_e_ in mg L^−1^ stand for CR initial and equilibrium concentrations, V represent solution volume in L, while m denotes NRPA amount used in mg. Percentage removal (R%) of CR was obtained thus:

2 $$ R(\% ) = \frac{{C_{o} - C_{e} }}{{C_{o} }} \times 100 $$

The experiments were performed in triplicate and the average values was reported.

### Desorption study

To assess the reusability of NRPA, adsorption cum desorption experiments were performed in six cycles using the already utilized adsorbent. Briefly, the desorption experiments were done by adding 15 mL solution of 0.1 M HCl to already used NRPA and placed on orbital shaker for agitation for 60 min. The content was filtered, and the CR concentration left in the filtrate was measured using spectrophotometer. The NRPA was used for another five cycles after each adsorption after thoroughly washing it with distilled water.

### Adsorption isotherms

Initial and equilibrium CR concentration relationship was examined using four common adsorption isotherms of two-parameters (Dubinin–Radushkevich (D-R), Langmuir, Temkin, and Freundlich), and three-parameters isotherms (Sips and Redlich-Peterson (R-P)). A Microsoft Math Scientist version 3.0. was deployed to predict the acceptability, while the suitability of the models was estimated from the values of the correlation coefficients, R^2^. The Langmuir, D-R, Temkin, and Freundlich isotherm are listed in Eqs. (3–6)^1–4,19–25^:

3 $$ Q_{eq} = \frac{{Q_{\max } bC_{e} }}{{1 + bC_{e} }} $$

4 $$ Q_{e} = Q_{s} e^{{ - \beta \varepsilon^{2} }} $$

5 $$ Q_{e} = \frac{RT}{{b_{T} }}\ln a_{T} C_{e} $$

6 $$ Q_{eq} = K_{F} C_{e}^{1/n} $$

Langmuir isotherm assumed that the entire surface where the adsorption process takes place is uniform and no interaction between the adsorbed molecules^20^. Q_max_ denotes maximum uptake of CR (mg g^−1^), C_*e*_ (mg g^−1^) denotes CR concentration at equilibrium and b represents constant of Langmuir in L mg^−1^.

Separation factor (R_L_) from the Langmuir model is given as^23^:

7 $$ R_{L} = {\raise0.7ex\hbox{$1$} \!\mathord{\left/ {\vphantom {1 {\left( {1 + bC_{0} } \right)}}}\right.\kern-0pt} \!\lower0.7ex\hbox{${\left( {1 + bC_{0} } \right)}$}} $$

Isotherm of the Freundlich assumed a heterogeneous surface in which adsorbed molecules interact together^1,2,6^. The Freundlich constants of *n* and *K*_F_ stand for adsorbent intensity and adsorption capacity respectively^1,2,6^. Temkin isotherm assumption is such that the surface coverage of the adsorption is a function of the free energy^23^. The parameters ‘a_T_’ and ‘b_T_^’^_’_ denote binding energy in L mg^−1^ and adsorption heat process respectively, T stands for temperature (K), while R denotes the ideal gas constant (8.314 J mol^−1^ K^−1^). D–R isotherm can be utilized for the classification of adsorption processes into either physisorption or chemisorption^23^. Saturation capacity in mol g^−1^ is given as *Q*_*s*_, while Polanyi potential (ε) is expressed as:

8 $$ \varepsilon = \ln \left( {1 + {\raise0.7ex\hbox{$1$} \!\mathord{\left/ {\vphantom {1 {C_{e} }}}\right.\kern-0pt} \!\lower0.7ex\hbox{${C_{e} }$}}} \right) $$

The expression β in mol^2^ J^−2^ denotes the adsorption process mean free energy (E) (kJ mol^−^)^19,23^. The mathematical expression is given as:

9 $$ E = \left( {2\beta } \right)^{ - 0.5} $$

When E is lesser than 8 kJ mol^−1^, it is physisorption, but when E is between 8 and 16 kJ mol^−1^, it denotes chemisorption^19^.

The three-parameters isotherm models of R–P and Sips are represented as^26,27^:

10 $$ Q_{e} = \frac{{Q_{o} C_{e} }}{{1 + K_{R} C_{e}^{g} }} $$

11 $$ Q_{e} = \frac{{Q_{s} \left( {K_{s} C_{e} } \right)^{{\beta_{s} }} }}{{1 + \left( {K_{s} C_{e} } \right)^{{\beta_{s} }} }} $$

where *K*_*R,*_ in L mg^−1^ stands for the R–P constant; g denotes the R–P exponent that vary between 0 and 1. Langmuir isotherm equation is predicted if β is 1 and when β equals 0, the R-P reduces to Freundlich isotherm equation^24^^,^^25^. Ks represents the Sips isotherm equilibrium constant in L mg^−1^, Q_s_ represents adsorption capacity in mgg^−1^. Values of exponent (β_s_) assume Langmuir equation when β_s_ = 1, and becomes Freundlich isotherm when either C_e_ or K_S_ tends towards 0^24,25^.

### Adsorption Kinetics studies

Mechanisms of adsorption involved in the uptake process was further explored by Intra-particle diffusion kinetic, pseudo-first-order (PFO), pseudo-second-order (PSO), and Elovich models as listed in Eqs. (12–15) below^1–4,19^:

12 $$ Q_{t} = K_{id} t^{0.5} + Ci $$

13 $$ Q_{t} = Q_{e} \left( {1 - e^{{ - k_{1} t}} } \right) $$

14 $$ Q_{t} = \frac{{k_{2} Q_{e}^{2} t}}{{1 + k_{2} Q_{e} t}} $$

15 $$ Q_{t} = {\raise0.7ex\hbox{$1$} \!\mathord{\left/ {\vphantom {1 \beta }}\right.\kern-0pt} \!\lower0.7ex\hbox{$\beta $}}\ln \left( {\alpha \beta *t} \right) $$

The rate constant relating to intraparticle diffusion in mg g^−1^ min^−0.5^) is given as *K*_*id*_, while the intercept which defines the adsorbent surface thickness is given as C_i_. Given that Q_e_ and *Q*_*t*_ stand for the pollutant concentrations adsorbed in mg g^−1^ at equilibrium and time *t (*min), *k*_*1*_ and *k*_*2*_ represent adsorption rate constants in min^−1^ and gmg^−1^ min^−1^ for the first- and second-order respectively, *t* denotes contact time (min), α and β are the Elovich constants representing the initial adsorption rate (mg g^−1^ min^−1^) as well as the desorption constant (g mg^−1^) respectively.

### Statistical test

Least square fit was the basis for best fit, it becomes imperative therefore to compare distribution error for the acceptability of the best model fit. For this purpose, the root mean square error (RMSE) and sum square error function (SSE) were engaged to confirm the best fit kinetic and isotherms models fit as indicated in Eqs. (20, 21)^24,25^:

16 $$ SSE = \sum\limits_{i = 1}^{N} {\left( {Q_{(\exp )} - Q_{(cal)} } \right)^{2} } $$

17 $$ RMSE = \sqrt {\frac{{\sum\limits_{i}^{N} {\left( {Q_{(\exp )} - Q_{(cal)} } \right)^{2} } }}{N}} $$

The parameters numbers in the model and the number of data points are given as* P* and *N* respectively. The lower the values of the distribution errors, the more acceptable the model.

### Thermodynamics studies

The spreading of the molecules of the solutes in solution at equilibrium can be deploy to determine constant of equilibrium, *K*_*d ,*_ expressed as^19,27,28^:

18 $$ K_{d} = \frac{{Q_{e} }}{{C_{e} }} $$

The free energy change (∆*G*°), enthalpy change (∆*H*°) and entropy change (∆S°) can be deduced from the equilibrium constant in the relationships below^1–3^:

19 $$ \Delta G^\circ = - RT\ln K_{D} $$

20 $$ \Delta G^\circ = \Delta H^\circ - T\Delta S $$

21 $$ \ln K_{d} = {\raise0.7ex\hbox{${\Delta S^\circ }$} \!\mathord{\left/ {\vphantom {{\Delta S^\circ } R}}\right.\kern-0pt} \!\lower0.7ex\hbox{$R$}} - {\raise0.7ex\hbox{${\Delta H^\circ }$} \!\mathord{\left/ {\vphantom {{\Delta H^\circ } {RT}}}\right.\kern-0pt} \!\lower0.7ex\hbox{${RT}$}} $$

## Results and discussions

### Characterization of NRPA powder

**Figure 1:**
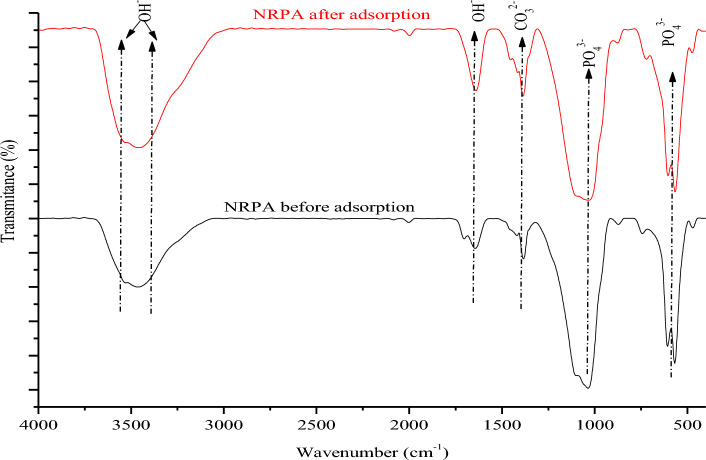
FT-IR analysis of the nano round polycrystalline adsorbent (NRPA) obtained from chicken bone before and after adsorption.

**Figure 2:**
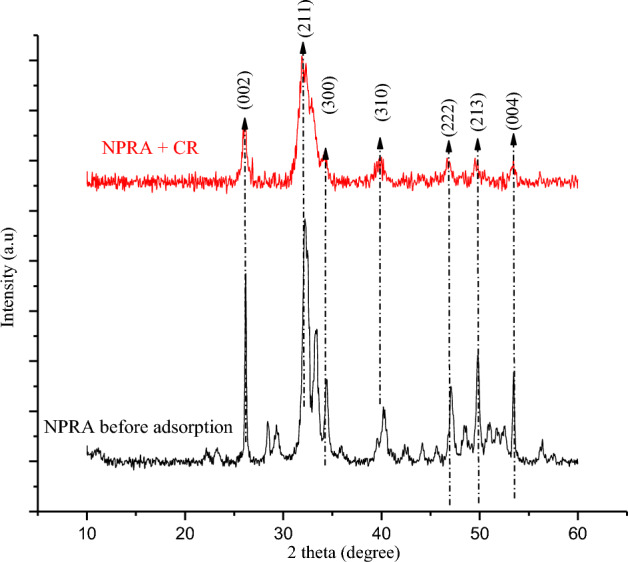
XRD analysis of the nano round polycrystalline adsorbent (NRPA) obtained from chicken bone before and after adsorption of CR.

The chicken derived nano-polycrystaline adsorbent powders were examined using FT-IR before and after CR adsorption as represented in Fig. 1. The NRPA powder showed major stretching vibrations between 1025 and 1055 cm^−1^ indicating the presence of phosphate^19^, O–P–O bending (v4) were noticed at 567 and 610 cm^−1^. The bands appearing at 887 and 1465 cm^–1^ designate the presence of carbonate group. The O–H stretch was noticed between 3365 and 3664 cm^−1^. After the adsorption of CR, the wavelength of the NRPA shifted with new peaks appearing in the ranges of 1023–1045 cm^−1^, 885–1462 cm^−1^ and 3360–3655 cm^−1^ indicates that phosphate, carbonate, and hydroxyl functional groups were involved in the adsorption process. These shifts in bands position is a confirmation of NRPA interaction with the dye molecules. Figure 2 represents the XRD patterns of the NRPA adsorbent before and after the uptake of CR molecules. As depicted, the peaks of the NRPA powder were identified by JCPDS file no. 090–432 and corresponding main planes of hydroxyapatite were confirmed at (002) and (211) planes. The poly-round crystalline nature of NRPA was confirmed by the occurance of many sharp peaks. Oher standard peaks assigned to hydroxyapatite powder were equally detected. Reductions in peaks, peaks broadening coupled with reduction in peaks intensity were observed after the uptake of CR which further confirmed the interaction between the NRPA adsorbent and the CR molecules.

**Figure 3:**
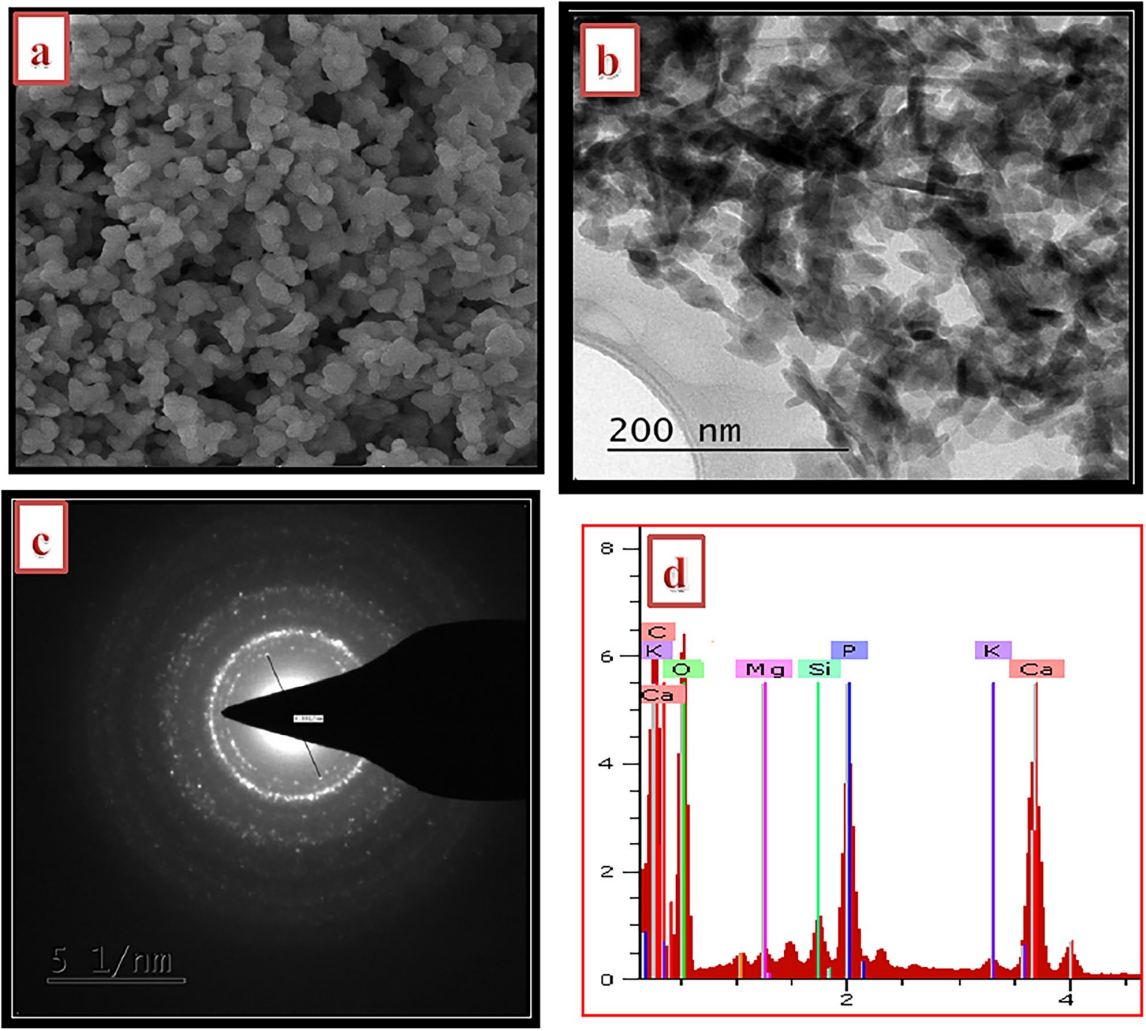
(**a**) SEM image, (**b**) TEM image, (**c**) SAED and (**d**) EDX analyses of the nano round polycrystalline adsorbent (NRPA) obtained from chicken bone.

SEM image of chicken bone derived adsorbent is shown in Fig. 3a with morphology of round shapes and particle size of between 10 and 44 nm. The round shape morphology was equally confirmed by the TEM analysis (Fig. 3b), while the selected area electron diffraction (SAED) also corroborated the polycrystalline nature of the apatite with spotted sharp and continuous rings system (Fig. 3c). The EDX revealed that oxygen, phosphorus and calcium are the major composition of the chicken bone with some trace amount of elements such as carbon, silicon, magnesium and potassium thus indicating the biogenic nature of the adsorbent (Fig. 3d).

### Equilibrium study

#### Contact time and CR initial concentrations

**Figure 4:**
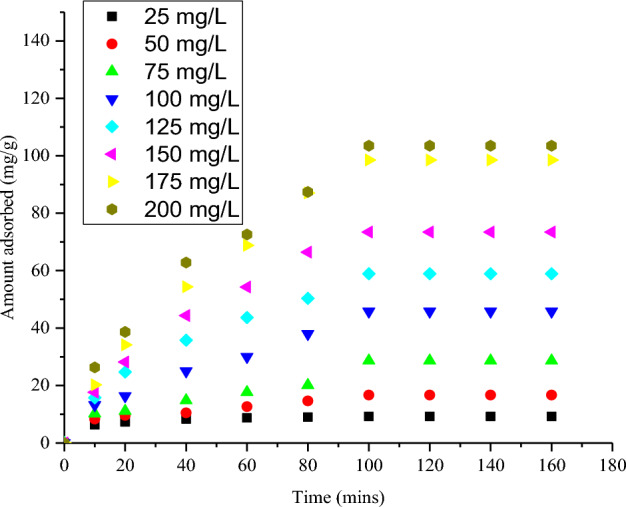
Function of contact time and CR concentration for the uptake of CR dye by nano round polycrystalline adsorbent (NRPA).

**Figure 5:**
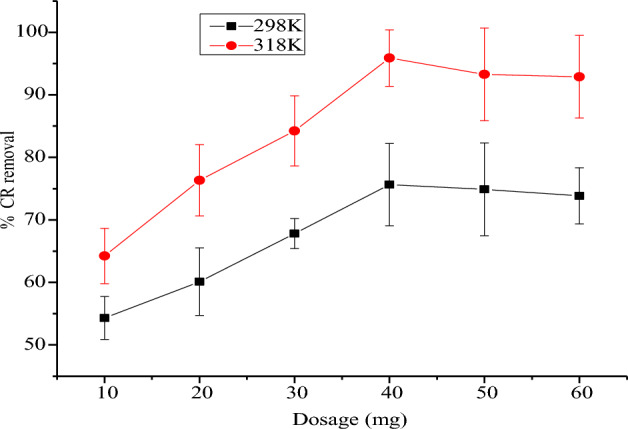
Function of nano polycrystalline adsorbent dosage for the uptake of CR dye by nano round polycrystalline adsorbent (NRPA).

The maximum uptake of CR as it relates to contact time is depicted in Fig. 5. As seen in the plot, CR dye removal rose progressively with the contact time pending the attainment of equilibrium point at 100 min. An increase in capacity of adsorption was noticed from 6.35 to 10.27 mg g^−1^ on altering contact time from 10 to 100 min at 25 mg L^−1^ CR concentration and from 26.33 to 103.47 mg g^−1^ at 200 mg L^−1^. After attaining the highest adsorption ability at 100 min, the CR removal was noticed to uniformed with time. The initial enhancement of the uptake rate of the dye can be attributed to the accessibility of excess adsorption sites of the adsorbent which gradually becomes occupied as the uptake time increases and as such, the adsorbent ability gradually became exhausted and only few vacant sites remained. Similar observation was documented by Wanyoni et al.^29^ for Congo red dye removal. Figure 4 showed that CR concentration equally affects the uptake of the contaminant. The uptake potency of NRPA adsorbent was enhanced when CR concentration was increased. Adsorbate concentration increase enhanced mass gradient transfer of CR molecules in aqueous phase and the solid phase which is the driving force for the transportation of CR particles from aqueous solution onto NRPA surface^30^.

#### The role of NRPA dose

Figure 5 reveals the role of NRPA dosage on the consumption of CR at two diverse temperatures of 298 K and 318 K respectively. The uptake efficacy increased from 53.44 to 78.65% at 298 K and from 66.34 to 95.30% at 318 K when the NRPA dosage was raised from 10 to 40 mg respectively. This phenomenon can be rationalized on the accessibility of much sites as a result of large surface area which enhanced the uptake process^19,29^. But, further rise in the dosage of NRPA resulted in lower uptake of CR owing to aggregation of CR particles on the adsorption sites which reduces the available sites for the uptake of the CR dye^19,29^.

#### Effect of Congo red dye pH

**Figure 6:**
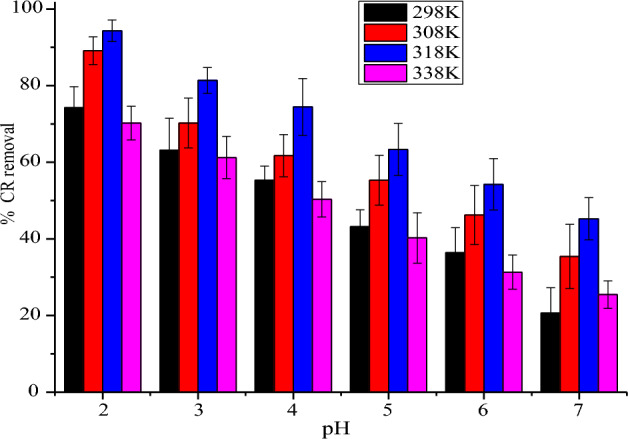
Function of pH on the uptake of CR dye by nano round polycrystalline adsorbent (NRPA).

The role that pH plays on CR adsorption was explored in temperature range of 298 K to 338 K (Fig. 6). Maximum adsorption percentage removal of CR at 298 K, 308 K, 318 and 338 K were recorded at maximum pH of 2 to be 77.40%, 87.40%, 94.70% and 70.33% respectively. Solution pH is very vital in sorption process owing to the fact that the chemistry of both the adsorbent as well as the adsorbate medium can be greatly affected. The pH_ZPC_ of NRPA was deduced to be 3.5 and below this, NRPA surface is anticipated to be positively charged. Congo red being an anionic dye is expected to exist as negative charge in acidic pH and this enhances CR adsorption onto the positively charged NRPA surface^2,7,31^. Congo red is zwitterion molecular and an acid–base indicator as a result of the sulfonated group (–SO_3_^−^) and amine group (–NH_3_^+^). At strong acid condition (pKa < 5), Congo red colour turns blue and the concentration of H^=^ in the solution is high and when the adsorbent surface at lower pH adsorbed H^+^, it becomes positively charge which enhances the adsorption of the negatively charged sulfonated group of the Congo red via electrostatic attraction, which enhances greater performance of the adsorbent. However, with increase in pH value above the pH_ZPC_, deprotonation of carboxyl, phosphate and hydroxyl functional groups available on the NRPA surface occurs which led to a repulsive attraction amid the sites of the adsorbent which are negatively charged as well as the anionic dye. According to Ogundiran et al.^30^, optimum pH of 2.0 was reported for CR uptake by unmodified snail shell and acid modified snail shell with maximum percentage removal of 65% and 77% respectively. It was concluded that the electrostatic interaction between the adsorbent surface which is positively charged at lower pH and various functional groups of CR molecules which are negatively charged were responsible for the high uptake of CR at lower pH and that at higher pH, the adsorption of the anionic dye decreases due to deprotonation on the surface of the biomass^30^.

Kumar et al.^32^ also observed that the reaction of molecules of CR with polyaniline@MoS_2_-based organic–inorganic nanohybrid surface decreases with rise in solution pH, which was due to the electrostatic relationship between protonated Pani@MoS_2_ and the negatively charged molecules of CR which took place in the acidic medium, thus enhancing CR molecules adsorption. Similarly, Munagapati and Kim^33^ reported that during the uptake of molecules of CR by CABI nano-goethite adsorbent, it was observed that the maximum sorption capacity of CABI nano-goethite was 137.7 mg g^−1^ at pH 3.0. They opined further that at this pH, the amount of hydrogen ions in the medium is much and that the adsorbent surface gains positive charge which resulted in the high uptake noticed at pH 3.0. Vairavel et al.^34^ observed that the sorption percentage of CR reduced from 82.35 to 11.41% when pH rose from 6 to 12 and that the negatively charged CR molecules is as a results of excess hydroxyl ions in the medium which resulted in greater electrostatic repulsion amid the adsorbent surface and the molecules of dye at higher solution pH.

### Isotherms studies

**Figure 7:**
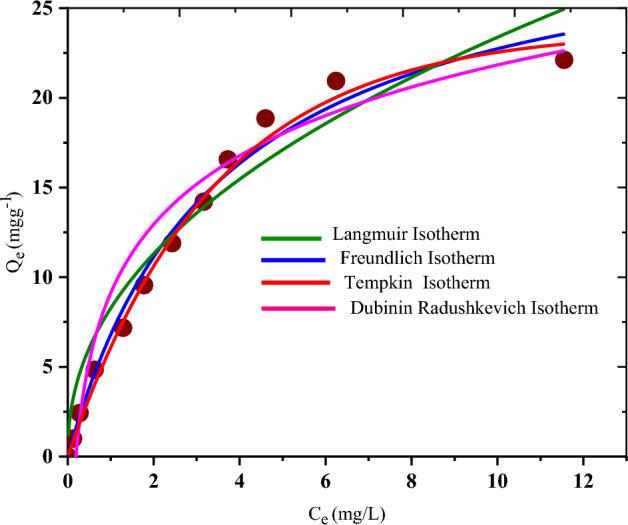
Two parameters isotherms for the adsorption of CR by nano round polycrystalline adsorbent (NRPA).

**Figure 8:**
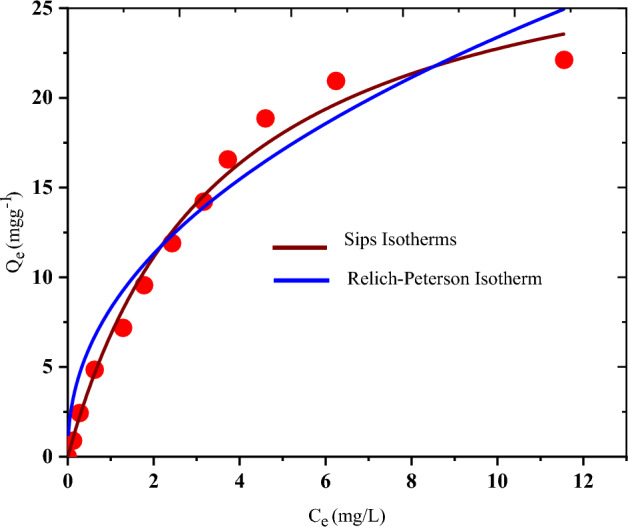
Three parameters isotherms for the adsorption of CR by nano round polycrystalline adsorbent (NRPA).

Figures 7 and 8 were used to deduce the constants of Freundlich, Temkin, D-R, and Langmuir as well as R-P and Sips isotherms respectively, while their constants are provided in Table 1. Correlation coefficient (*R*^*2*^) deduced are 0.996, 0.987, 0.977, 0.994, 0.984 and 0.986 for Langmuir, Freundlich, Temkin, D–R, Sips and R–P isotherms respectively and on the strength of the *R*^2^, the root mean square error (RMSE) as well as the sum square error function (SSE) values, the Langmuir isotherm performed better and thus governed the adsorption data. Therefore, relying on Langmuir assumptions, CR dye particles binding onto NRPA surface is monolayer adsorption with the receptor sites occurring on a homogeneous surface^30^. From the combined values of n from Freundlich and separation factor (R_L_) from Langmuir models, the adsorption of CR by NRPA is favourable^1,2,7^. The maximum adsorption capacities derived from Freundlich, Langmuir, Temkin, D–R, Sips and R–P isotherms are 44.562 mg g^−1^ mg L^−1/2^, 98.216 mg g^−1^, 26.327 mg g^−1^, 79.355 mg g^−1^, 62.103 mg g^−1^ and 49.205 mg g^−1^. Value of E obtained from D-R isotherm is 0.595 kJ mol^−1^ and this is smaller than 8 kJ mol^−1^ and as such, CR adsorption onto NRPA surface is said to be physical in nature^1^. The adsorption data were further examined using three parameters’ isotherms of R–P and Sips and the results obtained revealed good fit with both isotherms which combines the features of Freundlich and Langmuir models. When the adsorption capacity of NRPA was adjudged with further adsorbents in literature as indicated in Table 4, NRPA demonstrated higher adsorption affinity towards CR. The results further showcase chicken bone derived nano round polycrystalline adsorbent as a hopeful adsorbent in the elimination of CR from contaminated water.

**Table 1:** Isotherm parameters of NRPA in the adsorption of CR.

Isotherms	Parameter	CR
Langmuir	Q_max_ (mg g^−1^)	98.216
	b (L mg^−1^)	0.228
	R_L_	0.376
	R^2^	0.996
	%SSE	0.04
	RMSE	0.02
Freundlich	K_F_ ((mol g^−1^) (mol L^−1^)^−1/n^	44.562
	1/n	0.116
	R^2^	0.987
	%SSE	0.13
	RMSE	0.35
Temkin	a_T_ (L mg^−1^)	0.2422
	b_T_	26.3271
	R^2^	0.9770
	%SSE	0.25
	RMSE	0.33
Dubinin–Radushkevich	Q_s_ (mg g^−1^)	79.355
	β (mol J^−1^)^2^	3.17
	E (kJ mol^−1^)	0.595
	R^2^	0.994
	%SSE	0.13
	RMSE	0.22

### Kinetic studies

**Figure 9:**
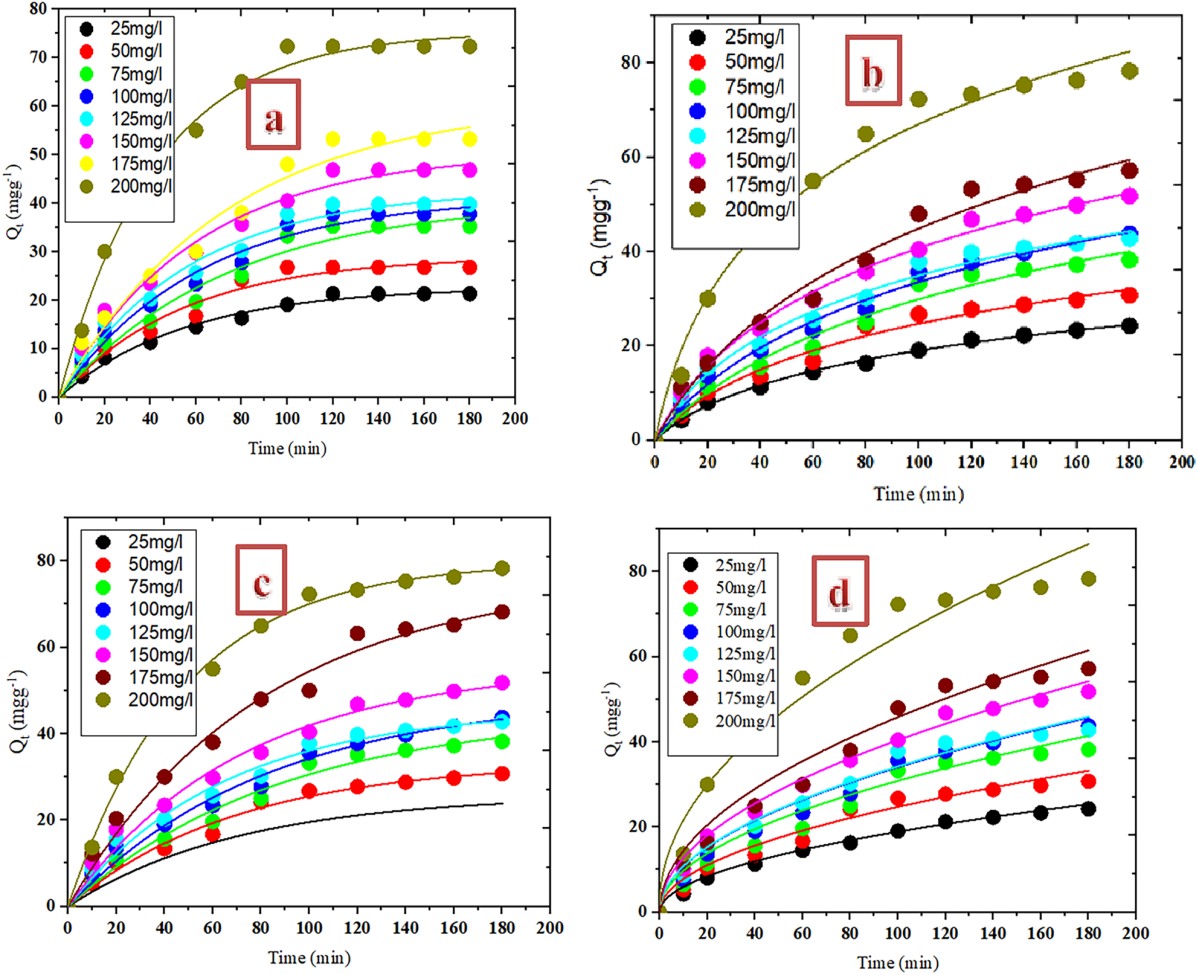
Kinetics models of (**a**) pseudo first order, (**b**) pseudo second order, (**c**) Elovich and (**d**) Intra particle diffusion for the adsorption of CR by nano round polycrystalline adsorbent (NRPA).

The resulting kinetic constants as well as the correlation coefficients (R^2^) so evaluated from Fig. 9 through a non-linear regression are provided in Table 2. Values of R^2^ derived from PFO kinetic model ranges between 0.987 and 0.998 and they are greater than the values of PSO model. In a similar vein, the experimental values of Q_e_ estimated from PSO are in better agreement with the theoretical Q_e_ values and this further suggests the suitability of this model in describing the uptake of CR by the NRPA. The acceptability of the first-order kinetic model for superior uptake of CR dye was further confirmed by RMSE and SSE which both have lower values when judged with RMSE values of PSO model. This implies that the mechanism of CR dye adsorption by NRPA active proceeds via physisorption process. Intra-particle diffusion values of K_id_ increased with CR concentration. Also, R^2^ values obtained ranged from 0.947 to 0.988 thus suggesting that the elimination of CR by NRPA molecules is described by this kinetic model. With this, it inferred that the sorption process of CR by NRPA can proceed via three stages: stage one encompasses the movement of the CR molecules from the CR media to the film surrounding NRPA surface and this is referred to as the film diffusion, second stage is diffusion of the CR particles from the surface onto NRPA pores and this is called pore or intra-particle diffusion, while the third step takes place when the CR particles are adsorbed onto the receptive sites of NRPA^19,35^.

**Table 2:** Kinetic parameters of NRPA in the adsorption of CR.

	C_o_ (mg L^−1^)	25	50	75	100	125	150	175	200
Pseudo-first order	*Q*_*e* exp_ (mg g^−1^)	9.270	16.680	28.650	45.780	58.930	73.440	98.530	103.470
	*Q*_*e* cal_ (mg g^−1^)	10.260	15.830	29.030	44.530	57.170	74.210	97.250	104.360
	*k*_*1*_ (min^−1^)	0.418	0.542	0.570	0.651	0.752	0.836	0.854	0.888
	R^2^	0.997	0.998	0.995	0.987	0.998	0.988	0.998	0.996
	%SSE	0.032	0.015	0.004	0.008	0.009	0.003	0.004	0.003
	RMSE	0.096	0.115	0.117	0.126	0.128	0.462	0.573	0.588
Pseudo-second order	Q_e cal_ (mg g^−1^)	13.838	20.600	32.720	50.734	62.076	87.680	96.540	115.103
	*k*_*2*_ (g mg^−1^ min^−1^)	0.204	0.299	0.347	0.364	0.588	0.576	0.747	0.763
	R^2^	0.896	0.922	0.889	0.936	0.975	0.967	0.878	0.983
	%SSE	0.149	0.071	0.043	0.033	0.016	0.058	0.006	0.034
	RMSE	0.106	0.122	0.145	0.179	1.433	1.723	2.331	2.127
Elovich	α (mg (g min)^−1^)	0.166	0.196	0.254	0.772	0.939	1.208	1.475	1.643
	β (g mg^−1^)	0.115	0.172	0.208	0.278	0.569	0.643	0.669	0.865
	R^2^	0.988	0.986	0.989	0.996	0.996	0.988	0.991	0.997
Intra-particle diffusion	*K*_*1d*_ (mg g^−1^ min^−0.5^)	0.219	0.370	0.558	0.763	1.454	1.705	2.359	4.563
	C_1_(mg g^−1^)	− 0.356	− 0.400	− 0.872	− 1.290	1.258	2.290	5.875	7.358
	R^2^	0.984	0.955	0.978	0.947	0.988	0.976	0.987	0.995

### Thermodynamics studies

**Figure 10:**
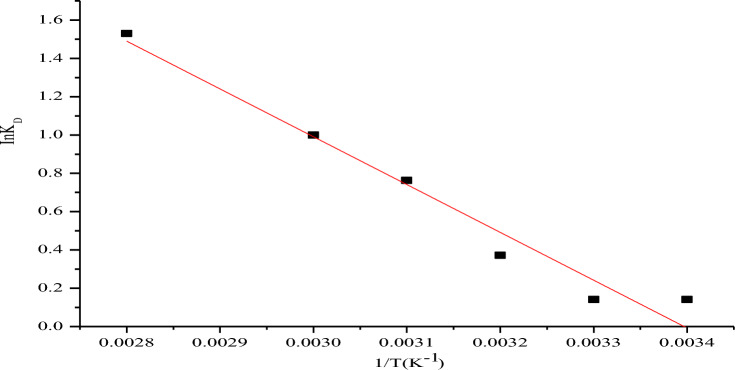
Plot of thermodynamic parameters for the adsorption of CR by nano round polycrystalline adsorbent (NRPA).

Thermodynamics constants were obtained from Fig. 10 with values provided in Table 3. As depicted, the entire ΔG° values which ranges from − 359.248 to − 4459.68 kJ mol^−1^ were all negative at different temperatures tested thereby suggesting that the uptake of CR by NRPA surface is spontaneous process. As the temperature increases, ΔG° values become more negative which is an indication that the uptake of CR by NRPA is facilitated at higher temperatures (Table 4). The physical nature of CR adsorption by NRPA was also established based on ΔG° values which are less than − 20 kJ mol^−133^. The value of ΔH° obtained is 5.280 kJ mol^−1^ which suggests that CR adsorption by NRPA to be endothermic process and also physical in nature^36,37^. Gupta *et al*^36^ observed that adsorption is said to be physical process when ΔH° < 25 kJ mol^−1^ but chemical when ΔH° > 40 kJ mol^−1^. Value of ΔS° obtained is 16.403 kJ mol^−1^ K^−1^ which indicates a rise in the gradation of haphazardness at NRPA/CR boundary of the adsorption process^37^.

**Table 3:** Thermodynamic parameters of NRPA in the adsorption of CR.

T (K)	ΔG (J mol^−1^)	ΔH (kJ mol^−1^)	ΔS (J mol^−1^ K^−1^)
298	− 35.248		
308	− 1.84		
318	− 2.56	5.280	16.403
328	− 3.75		
338	− -459.60

**Table 4:** Adsorption Capacities of various Adsorbents with NRPA in the adsorption of CR.

Adsorbents	Q_e_ (mg g^−1^)	References
Coffee husks powder	38.64	Vairavel et al.^34^
Mango leaf	4.49	Bello et al.^38^
Cabbage waste powder	2.313	Wekoye et al.^39^
FexCo_3_-xO_4_ nanoparticles	128.6	Liu et al.^9^
soybean curd xerogels	69.90	Zhang et al.^10^
Activated carbon coffee waste	90.00	Lafi et al.^13^
Tunics of the corm of the saffron	6.2	Dbik et al.^14^
Kaolin	5.94	Meroufel et al.^40^
Apricot stone activated carbon	32.85	Abbas and Trari^41^
Luffa cylindrica cellulosic fiber	17.39	Gupta et al.^42^
Cationic surfactant modified wheat straw	71.2	Cheng et al.^43^
Pani@MoS_2_	70.920	Kumar et al.^32^
PVC@GN–Pani–NH4OH	26.315	Kumar et al.^44^
PVC@GN–Pani–HC	31.250	Kumar et al.^44^
PVC@GN–Pani–CTAB	40.000	Kumar et al.^44^
NRPA	98.216	This study

### Mechanism of Congo red uptake by NRPA

**Figure 11:**
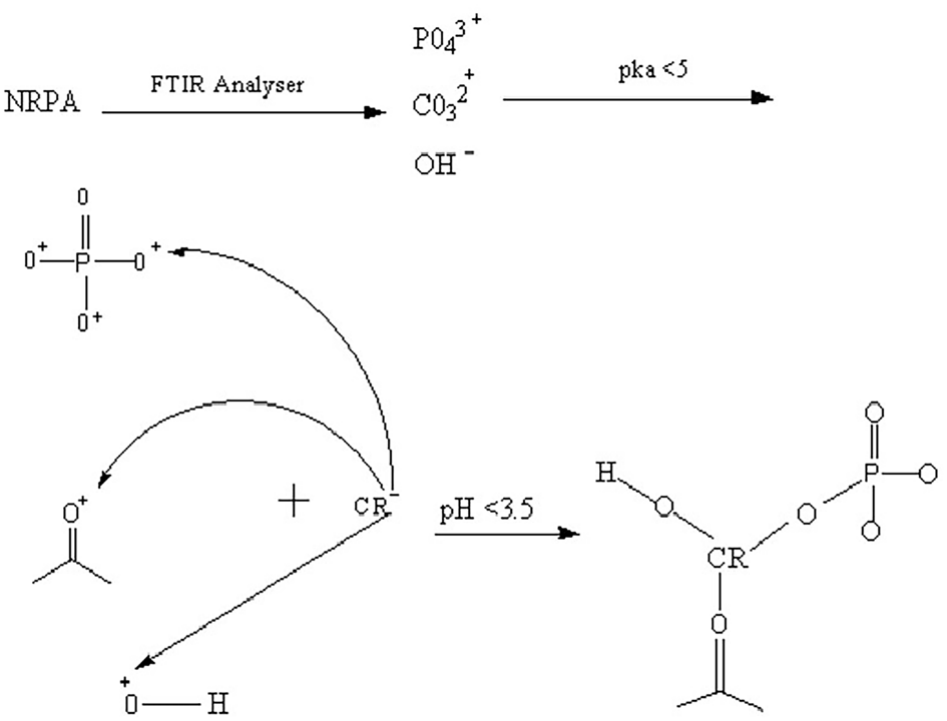
Schematic representation of adsorption mechanism of CR by NRPA.

Numerous mechanisms of adsorption are available to further elucidate the interactions which exist between the adsorbate (CR) and the adsorbent (NRPA) among which are ion-exchange, chemisorption, precipitation, complexation, electrostatic attraction, and physisorption^23^. The FT-IR study of NRPA showed the presence of CO_3_^2-^, O–H and PO_4_^3−^ which could become positively charged when the solution of CR is protonated at acidic pH and thereafter reacts with negatively charged CR through strong electrostatic attraction mechanism. This was corroborated further from the value of the pH_ZPC_ of NRPA which was obtained to be 3.5 and as such, NRPA surface is anticipated to be positively charged below this pH_ZPC_ value. Thus, Congo red being an anionic dye in acidic pH medium can easily adhered to the positively charged NRPA surface. According to Zhongxin et al.^45^, the sorption of Congo red by orange peel biochar modified with CTAB is electrostatic attraction and this is caused by the positively charged mango peel biochar modified with CTAB and Congo red which negatively charge is responsible for the adsorption. Maria et al.^19^ opined that adsorption mechanism of Congo Red dye by fly ash can be described through strong electrostatic attraction between fly ash surface which is mainly positive and CR molecules which is negative charged. They stressed further that functional groups such as -OH and Si–O–Si present on the adsorbent surface were responsible for the formation of electrostatic attractions with CR molecules. Using the values of correlation coefficients and the predicted values for qe and the experimental ones, Khaoula et al.^46^ proved that the adsorption of CR by pine bark is controlled by chemisorption mechanism. Their finding revealed that the predominant functional groups presence on the surface of pine bark at acid pH are positive, whereas, since the isoelectric point of Congo red is 3, the molecular structures is expected to be negative when the pH is greater than 3 which subsequently resulted in electrostatic attraction mechanism. Figure 11 shows the schematic representation of adsorption mechanism of CR by NRPA.

### Desorption study

**Figure 12:**
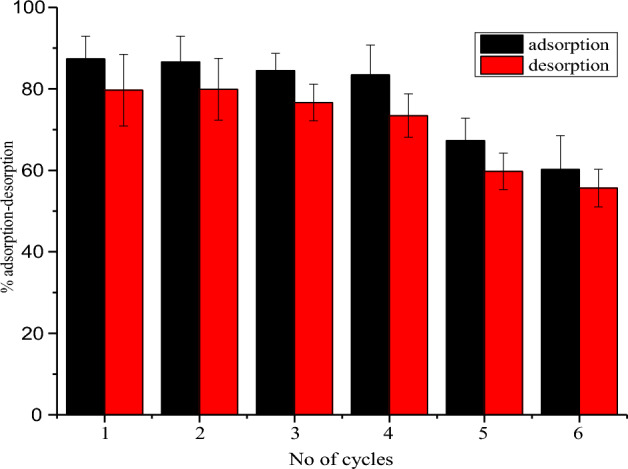
Adsorption–desorption study.

The results obtained from desorption experiments with HCl as eluting agent is shown in Fig. 12. It was revealed from the results that 0.1 M solution of HCl clearly desorbed the adsorbate from the surface of the adsorbent after each successive experiment. It was observed that the NRPA adsorption–desorption efficiency for CR was almost constant for the first three cycles with desorption efficiency of 83.88% after the fourth cycle. However, above the fourth cycles, the percentage adsorption decreased sharply indicating reduction in the adsorption ability of the adsorbent. The results clearly shows that the NRPA can be use more than one time and thus, demonstrate it’s regeneration and economic importance.

## Conclusion

This work investigates the adsorption potency of chicken bone derive adsorbent for anionic Congo red dye removal. FT-IR, XRD, TEM, SEM, and pH_pzc_ analyzes were done to characterized the fabricated adsorbent. Raw data obtained were subjected to different adsorption isotherms with the Langmuir model showing better conformity. Optimum adsorption was attained at solution pH of 2.0, adsorbent dosage of 40 mg, contact time of 100 min and initial CR concentration of 200 mg L^−1^ respectively. Data from kinetics examinations showed that the uptake of CR by NRPA obeys PFO model. The maximum adsorption capacities derived from Langmuir isotherm is 98.216 mg g^−1^. Constants of ΔG° calculated ranges from − 359.248 to − 4459.68 kJ mol^−1^ were all negative at different temperatures tested thereby suggesting that the uptake of CR by NRPA surface is spontaneous process and endothermic nature. On the strength of the above, chicken bone derived adsorbent was found to be a suitable adsorbent in the uptake of anionic Congo red dyes from contaminated water.

## Data Availability

The datasets generated and/or analysed during the current study are not publicly available due to restrictions apply to the availability of these data by the Department of Chemical Sciences, Mountain Top University, which were used under license for the current study but are available from the corresponding author on reasonable request.
